# MECHANISMS UNDERLYING SLEEP AND DEVELOPMENT OF CHRONIC CONDITIONS IN OLDER ADULTS

**DOI:** 10.1093/geroni/igad104.1827

**Published:** 2023-12-21

**Authors:** Christopher Kaufmann, Soomi Lee, Christina McCrae

## Abstract

Mounting evidence suggests poor sleep is associated with the development of chronic conditions. Numerous mechanisms for these links have been proposed, including inflammation, metabolic function, and brain structure. In this symposium, we will present five studies focused on links between poor sleep and subsequent development of chronic disease across adulthood. Specifically, we will examine this relationship at multiple levels from the cellular to the clinical level with the ultimate goal of identifying potential mechanisms and consequences of links between poor sleep and chronic conditions. Paper 1 will identify within-person patterns of various sleep experiences and link them to incident chronic conditions over a 10-year period. Paper 2 will examine associations between sleep parameters, measured via wrist actigraphy, and levels of adipokines, which are adipose-derived cytokines associated with development of metabolic disease. In Paper 3, we will shift our focus to examine the association between poor sleep and inflammatory biomarkers, specifically C-reactive protein, circulating and lipopolysaccharide-stimulated cytokines. Paper 4 will assess the impact of poor sleep health on fear of falling and actual falls. Paper 5 will focus on the effects of poor sleep and arousal with cognition and brain structure, specifically within a population of patients with chronic pain. As Discussant, Dr. Christina McCrae will integrate these findings and suggest directions for future research. In summary, our symposium will elucidate biological and psychological mechanisms linking poor sleep to manifestation of chronic disease. Better understanding of these mechanisms will inform the development of interventions to promote successful aging.

